# Adverse effects of paternal obesity on the motile spermatozoa quality

**DOI:** 10.1371/journal.pone.0211837

**Published:** 2019-02-11

**Authors:** Georges Raad, Joseph Azouri, Kamal Rizk, Nina S. Zeidan, Jessica Azouri, Valérie Grandjean, Mira Hazzouri

**Affiliations:** 1 Azoury-IVF clinic, Mount Lebanon Hospital, Camille Chamoun Boulevard, Beirut, Lebanon; 2 Lebanese University, Faculty of Sciences 2, Fanar, Lebanon; 3 INSERM U1065, Centre Méditerranéen de Médecine Moléculaire (C3M), Team 10 “Control of gene expression “, Nice, France and University of Nice Sophia Antipolis, Faculty of Medecine, Nice, France; Universite Clermont Auvergne, FRANCE

## Abstract

Growing evidence suggests that paternal obesity may decrease male fertility potential. During infertility treatment with intra-cytoplasmic sperm injection (ICSI), a morphologically normal motile spermatozoon is injected into a mature egg, when possible. However, sperm motility and morphology per se do not reflect the sperm molecular composition. In this study, we aimed to assess the quality of motile spermatozoa in the context of obesity by analysing their conventional and molecular characteristics as well as their ability to promote early embryonic development. A prospective study was conducted on 128 infertile men divided into three groups: 40 lean, 42 overweight, and 46 obese men. Conventional sperm parameters (concentration, motility and morphology) and sperm molecular status (chromatin composition and integrity, 5-methycytosine (5-mC) and 5-hydroxycytosine (5-hmC) contents and oxidative stress level) were analysed on raw semen and/or on motile spermatozoa selected by density gradient or swim-up techniques. Morphokinetic analysis of the embryos derived from ICSI was performed using the Embryoviewer software. Our results showed that the motile sperm-enriched fraction from obese men exhibited higher levels of retained histones (p<0.001), elevated percentage of altered chromatin integrity (p<0.001), and decreased contents of 5-hmC (p<0.001), and 5-mC (p<0.05) levels as compared to that from lean men. Importantly, there were no statistically significant correlations between these molecular parameters and the percentages of morphologically normal motile spermatozoa. Regarding embryo morphokinetics, the CC1 (p<0.05) and CC3 (p<0.05) embryonic cell cycles were significantly delayed in the cleavage embryos of the obese group as compared to the embryos of the lean group. Our data is of particular interest because, besides demonstrating the negative impacts of obesity on motile spermatozoa molecular composition, it also highlights the possible risk of disturbing early embryonic cell cycles kinetics in the context of paternal obesity.

## Introduction

Obesity, as defined by excessive accumulation of adipose tissue, is a worldwide public health crisis [1–4]. It is one of the risk factors leading to the development of several pathologies, such as type 2 diabetes mellitus (T2DM), cardiovascular diseases, respiratory diseases and hypertension [5, 6]. Furthermore, several studies have documented the possible association between paternal obesity and male infertility [7, 8]. This is especially alarming given the high prevalence of obesity among young men of reproductive age [9, 10].

Male fertility can be explored through the assessment of the conventional semen parameters (e.g., semen viscosity, sperm motility and sperm morphology), the in-depth examination of the sperm molecular composition and through the analysis of the embryo developmental ability. Several independent studies have shown that obesity negatively affects conventional semen parameters and subsequently reduces the male fertility potential. For instance, it was demonstrated that obesity in fathers significantly increases the incidence of oligozoospermia and azoospermia [11], reduces the percentage of sperm with normal morphology [12, 13] and increases the percentage of sperm with fragmented DNA [12] in the ejaculated semen.

Additionally, various reports have indicated that paternal obesity may alter the molecular composition of spermatozoa, entailing adverse consequences on the health of the respective progenies (review in [14, 15]). Specifically, it has been suggested that the spermatozoa epigenetic components, such as DNA methylation, chromatin structure and noncoding RNAs (ncRNAs) are very vulnerable to excessive obesity [16–21]. Studies that addressed this biological question in human indicated that male obesity increases the percentage of sperm with decondensed chromatin [22], alters the sperm DNA methylation at specific genomic regions [23] and affects the expression of several ncRNAs in sperm cells such as piRNA, microRNA and fragmented tRNAs [20]. In this regard, several studies strongly indicate that epididymal microRNAs play an important role in the regulation of several gene networks involved in the function of the epididymis and gamete maturation [24–26]. There are also few studies showing alterations in the expression of epididymal microRNAs in sperm from obese men [20]. Altogether, these data indicated that obesity could adversely impact the sperm quality and increase the risk of transmission of abnormal epigenetic materials to the offspring [16, 20, 27, 28].

It should be noted that the majority of these reports have been performed on raw semen containing motile, non-motile, and dead spermatozoa. However, during natural conception, motile spermatozoa are separated from other semen fractions in the vagina and only few motile sperm can reach the site of fertilization [29, 30]. In parallel, during intra-cytoplasmic sperm injection (ICSI) one motile sperm with normal morphology is usually immobilized and injected into a mature egg [31]. Unfortunately, motile sperm with normal morphology may contain molecular alterations that could affect ICSI outcomes and future child health [31, 32]. Therefore, there is a need to assess the impact of paternal obesity on the molecular composition of motile spermatozoa.

In addition to the impact of obesity on conventional and molecular sperm parameters, several studies have investigated the effect of male obesity on human embryo, but the results are still debated [33–38]. Although some of them did not find any significant differences in the analysed embryologic parameters between obese and non-obese men [37, 39], others have highlighted a significant decrease in blastulation rate with increasing body mass index (BMI) [22, 40]. Furthermore, a recent meta-analysis indicated that paternal obesity reduces the rate of live birth per assisted reproductive technology (ART) cycle and increases the risk of facing a non-viable pregnancy [12]. Recently, time-lapse imaging technology emerged in the reproductive medicine field leading to a better understanding of early embryo development and to a better embryo selection [41, 42]. One of the most remarkable findings in the reports using an incubator with a time-lapse system was the detection of a possible relation between embryonic cell cycle kinetics and pregnancy outcomes after ICSI [43–46]. Therefore, it is of paramount importance to deeply analyse the effects of paternal obesity on the embryonic cell cycle kinetics.

In the light of these studies, the aim of the present report was to compare various molecular characteristics of motile spermatozoa selected by density gradient technique or swim-up procedure from lean, overweight, and obese men. Moreover, given the interplay between sperm molecular components and embryonic development, we sought to analyse the impact of increased paternal body mass index on pre-implantation embryo morphokinetics.

## Materials and methods

### Study population

**Fig 1:**
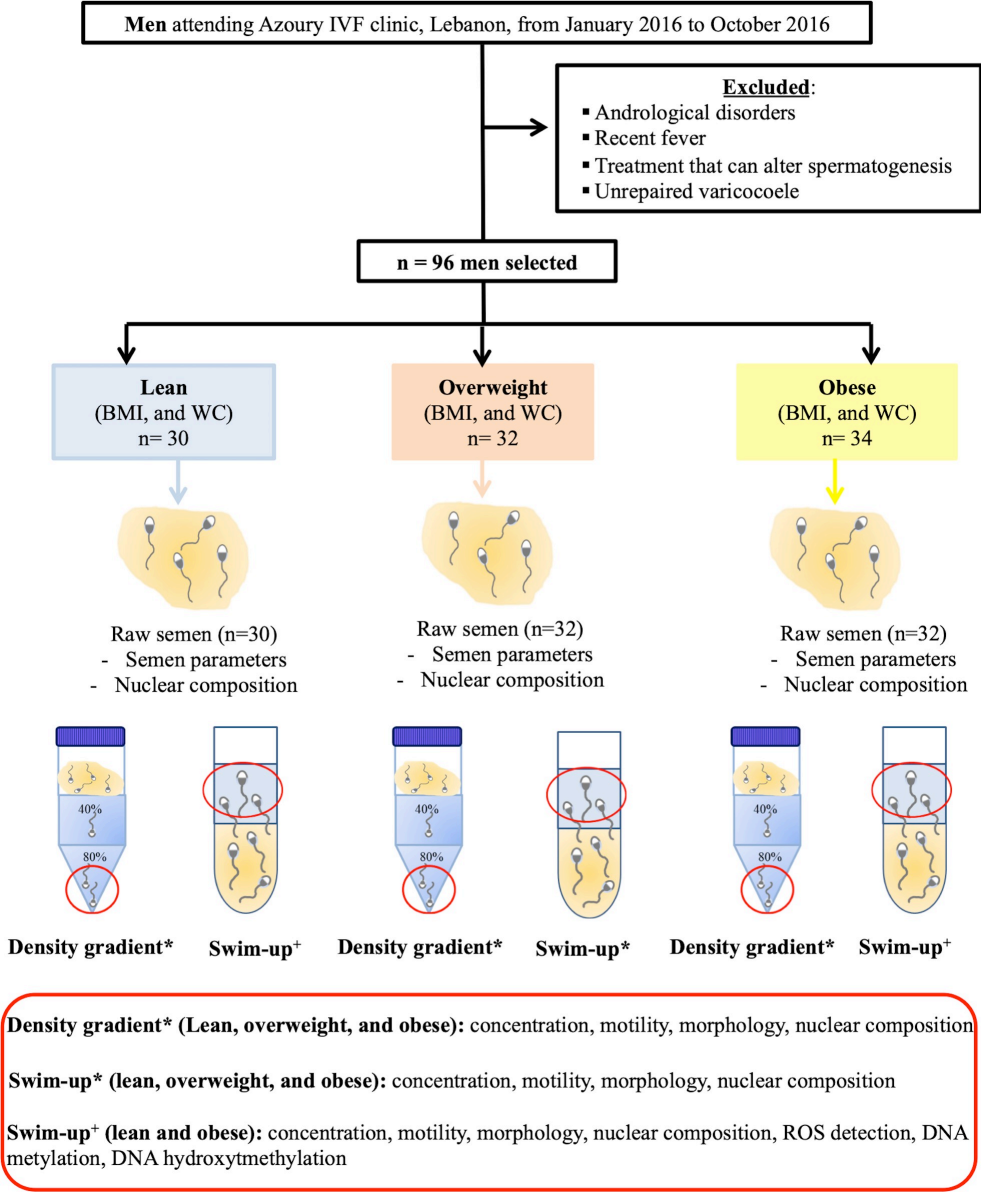
Study design for the selection of the motile sperm from the raw semen of lean, overweight, and obese men using the density gradient technique or the swim-up procedure. Each semen sample was equally divided into two aliquots. Each aliquot was addressed to one of the motile sperm selection techniques. The concentration, motility, morphology, and nuclear composition (histone retention and DNA integrity) were assessed on the raw semen and on the selected motile spermatozoa (in the red circles). In addition, the detection of the reactive oxygen species (ROS) and the level of the DNA methylation/hydroxymethylation were performed on the motile sperm derived from the swim-up procedure from lean and obese men.

**Fig 2:**
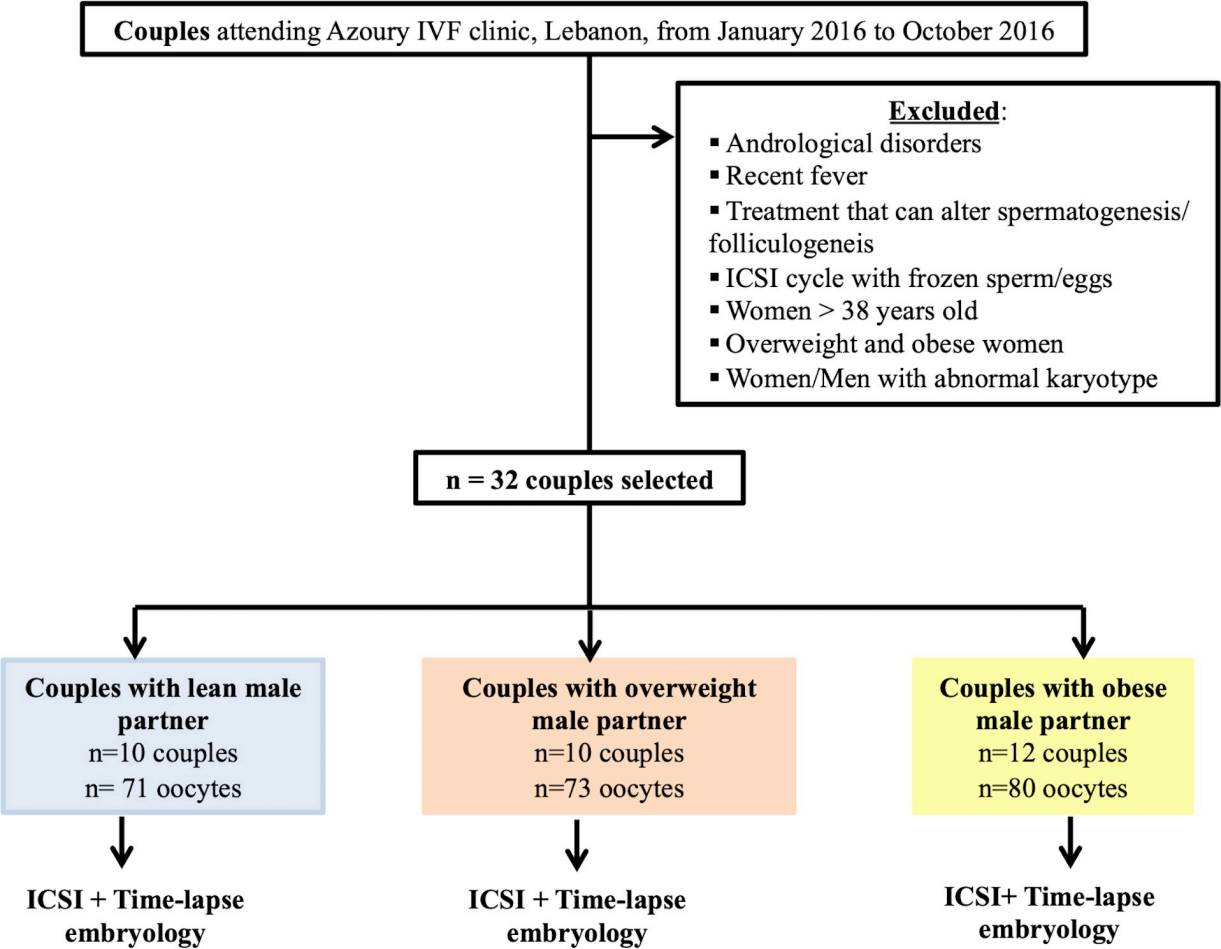
Study design for the analysis of the embryo morphokinetics from the three different groups. For this part of this study, all the semen samples were processed using the swim-up procedure.

Semen samples were obtained from 128 men attending the Azoury IVF clinic, fertility center at Mount Lebanon hospital—Hazmieh, Lebanon- between January 2016 and October 2016. On one hand, 96 semen samples were used to compare the impact of two sperm preparation techniques on the motile sperm characteristics, from lean, overweight and obese men (Fig 1). On the other hand, 32 semen samples were exclusively used for intra-cytoplasmic sperm injection (ICSI) (Fig 2). In-person interviews were conducted to complete a questionnaire about the age, the length of the sexual abstinence, current or previous disease status including urogenital ones, and habits such as smoking and alcohol intake. All participants suffering from andrological disorders, unrepaired varicocoele, recent fever, the moderate or heavy smokers (more than 21 cigarettes per day), the moderate or heavy alcohol consumers (more than 5 drinks per week), those undertaking any treatment that may alter spermatogenesis and those using a frozen sperm/eggs cycle for ICSI were excluded from the study (Fig 1). For the pre-implantation embryo morphokinetic analysis, data were collected from 32 couples undergoing ICSI cycles. Women were under 38 years-old at the time of oocyte collection [40] (Fig 2).

### Ethical approval

An informed consent was obtained from each participant before the participation in this clinical study. This project has received the approval of the ethical committee of Mount Lebanon Hospital.

### Anthropometric measurements

Anthropometric measurements were performed by trained staff. Weight was measured in kilograms using a weighing scale. Height was measured in centimetres. The body mass index (BMI) was calculated as weight in kilograms divided by the squared height in meters, and it was categorized as follows: 18<BMI<25kg/m^2^ (lean), 25≤BMI<30 kg/m^2^ (overweight), and BMI ≥30 kg/m^2^ (obese) [47].

Waist circumference (WC) was measured with a standardized tape measure, which was placed over the skin or light clothing while the participant was standing. Two measurements were usually taken followed by a third one when the difference between the first two measurements was 0.5 cm or above [47].

From these measurements, men were considered obese when their BMI was above 30 and their WC above 102 cm, overweight when their BMI was comprised between 25 and 30kg/m^2^ and their WC comprised between 90 and 102 cm, and lean when their BMI was under 25 and their WC under 90. It is important to note that during all the following procedures, the embryologists were blinded as to the origin of the sperm or embryos being analysed.

### Semen analysis

A semen sample was produced on-site by masturbation into a sterile plastic specimen cup. All subjects underwent semen analysis. The analysis of the semen parameters such as sperm concentration (x10^6^ sperm/ml), sperm motility and sperm morphology were performed according to the World Health Organization criteria 2010 [48].

### Isolation of the motile spermatozoa

For the 96 semen samples used to analyse the molecular composition of the motile spermatozoa derived from two sperm preparation techniques, an auto-controlled study was conducted. In this context, each semen sample was equally divided into two aliquots and each aliquot was addressed to the swim-up procedure or density gradient centrifugation (Fig 1). All samples were prepared using the same products, in the same laboratory and evaluated by 2 embryologists. Readings were averaged between the two evaluations after calculation of error [48]. For the 32 semen samples used for ICSI, the semen was exclusively processed using the swim-up procedure (Fig 2) for ethical reasons.

#### Motile sperm isolation using swim-up technique

From each semen sample, one aliquot was used to prepare the motile sperm—enriched fraction using the swim-up technique [49]. Briefly, one volume of semen was placed in a tube and was overlaid by one volume of culture medium (Sperm Medium, COOK medical, Australia) according to the manufacturer’s instructions. The tube was incubated for 45 min at 37°C under 5% CO_2_, 5% O_2_ and 90% N_2_. The supernatant was aspirated and transferred to an empty tube: replicate measurements of sperm concentration, sperm motility, and sperm morphology were performed according to the World Health Organization criteria 2010 [48] (Fig 1).

#### Motile sperm isolation using density gradient technique

A second semen portion from each semen sample was used to isolate the motile spermatozoa by the density gradient technique; 40 and 80% Silane-coated silica in gamete buffer (Gradient kit from COOK medical) according to the manufacturer’s instructions. Semen was layered onto the gradient and processed according to the manufacturer’s recommendations [48]. The pellet was collected: replicate measurements of sperm concentration, sperm motility, and sperm morphology were performed according to the World Health Organization criteria 2010 (Fig 1).

### Sperm morphology evaluation

In order to observe the sperm morphology, dried smears were stained using the Spermoscan Kit (RAL diagnostics). The staining was performed according to the protocol of the manufacturer. At least 200 sperms were counted and classified as having a normal or an abnormal morphology according to Kruger’s strict criteria; head defects: large or small, tapered, amorphous, vacuolated (more than two vacuoles or >20% of the head area occupied by unstained vacuolar areas), small or large acrosomal areas; mid-piece defects: bent, cytoplasmic residues; tail defects: coiled, multiple [48].

### Sperm vitality assessment

Sperm vitality was assessed using the eosin-nigrosin staining. The results were expressed as the percentage of stained or pink sperm after examination of 200 spermatozoa [48].

### Intracellular reactive oxygen species (ROS) detection

To evaluate the intracellular ROS levels in the sperm of lean and obese men, we performed the nitroblue tetrazolium test (NBT) (Fig 1). It is a direct test used to detect the reactive oxygen species within the spermatozoa [50]. A total number of 200 spermatozoa were scored, per smear, under 100x magnifications. The spermatozoa were scored as NBT^+^ cell (containing the dark formazan precipitate resulting from the interaction between the NBT and the intracellular ROS) or NBT^-^ cell (does not contain the formazan) [50].

### Sperm histone retention assessment

Sperm histone retention was evaluated using the aniline blue staining protocol described elsewhere [51], which discriminates between lysine-rich histones and arginine/cysteine-rich protamines. Sperm heads containing high percentage of histones were stained by blue colour and those with normal histones content did not take up the stain. The percentage of spermatozoa stained with aniline blue was determined by counting 200 spermatozoa per slide under bright field microscopy [51].

### Sperm chromatin integrity assessment

Chromatin integrity was assessed using the toluidine blue (TB) method [52]. The toluidine blue is a cationic dye. It can bind to the negatively charged phosphate residues of the DNA in the loosely packed chromatin and/or impaired DNA [53]. Two hundred randomly selected spermatozoa per sample were examined under high magnification. The cells were classified into two groups: dark violet cells (TB^+^ cells; abnormal chromatin structure) and light blue cells (TB^-^ cells; normal chromatin structure) [52].

### Sperm DNA extraction

Sperm pellets were subject to an osmotic shock and a flash freeze shock in liquid nitrogen for cellular lysis. In order to characterize DNA chemical modifications in the motile-sperm enriched fraction, the sperm derived from the supernatant of the swim-up technique was selected for DNA extraction. Each sample was thawed on ice and sperm DNA was subsequently extracted using a detergent-based lysis followed by an in-column purification using the QIAamp DNA Mini Kit (#51304; Qiagen, The Netherlands). DNA yields and quality were determined using the Nanodrop 2000 Spectrophotometer (#E112352; Thermo Scientific, Somerset, NJ).

### Sperm global DNA 5-methylcytosine (5-mC) and 5-hydroxymethylcytosine (5-hmC) measurements

We used Methylated DNA Quantification Kit (Colorimetric) (ab117128; abcam, USA) and Hydroxymethylated DNA Quantification kit (Colorimetric) (ab117130; abcam, USA) for the quantification of 5-mC and 5-hmC, respectively. These Enzyme-Linked Immunosorbent Assay (ELISA) analyses were performed according to the manufacturer's recommendations. The samples were read on an automated plate reader at 450 nm absorbance. The relative 5-methylcytosine (5-mC) and 5-hydroxycytosine (5-hmC) percentages were calculated using the following formula provided by the manufacturer:

$$5-mC\%=\frac{(Sample\;OD-Negative\;OD):S}{(Positive\;Control\;OD-Negative\;Control\;OD)x2*:2}x100\%$$

and

$$5-hmC\%=\frac{(Sample\;OD-Negative\;OD):P}{(Positive\;Control\;OD-Negative\;Control\;OD)x5*:2}x100\%$$

S and P are the amounts of input sample DNA in ng, and of input positive control in ng, respectively. 2* and 5* are factors to normalize 5-mC in the positive control to 100%, as the positive control contains only 50% of 5-mC and to normalize 5-hmC in the Positive Control to 100, as the Positive Control contains only 20% of 5-hmC, respectively.

### Ovarian stimulation, oocytes retrieval, and ICSI

In addition to the inclusion criteria cited above for men selection, we used the following criteria for the women: aging 38 years old or less, BMI between 18 and 25kg/m^2^, with normal karyotype and normal response to ovarian stimulation. All women in this study underwent a controlled ovarian stimulation with antagonist protocol (Fig 2). Recombinant human chorionic gonadotrophin (hCG) was administered when at least three follicles were >17mm. Oocytes retrieval was performed 36 hours post-hCG administration [40]. Freshly collected oocytes were cultured for 3 hours in Global fertilization medium (Life Global, Canada). ICSI was performed at 39 hours post hCG administration with spermatozoa selected from the motile sperm enriched fraction post swim-up technique. Then, the injected eggs were placed inside a pre-equilibrated embryoslide (Embryoslide, Vitrolife) containing 12 wells; each filled with 25 μl of Global medium (Life Global, Canada) and overlaid with 1.2 ml of culture oil (Life Global, Canada). The pre-equilibration step was performed overnight at 37°C under 5% CO_2_ [54].

### Embryo culture and evaluation of time-lapse images

Embryoslides containing the injected eggs were placed in the Embryoscope (Vitrolife) immediately after ICSI. They were cultured for 5 consecutive days without interruption at 37°C with 5% CO_2_, 5% O_2,_ and 90% N_2_. Images were acquired each 10 min for every embryo at seven focal plans (10 μm intervals). Embryo quality and morphokinetics were analysed using the Embryoviewer software (Vitrolife). The analyses allowed to determine the precise timing of pronuclei appearance (tPNa), pronuclei fading (tPNf), duration of the first cell cycle (CC1 = time to 2 cells (t2)-tPNf), second embryonic cell cycle (CC2 = time to 4 cells (t4)–t2), and third embryonic cell cycle (CC3 = time to 8 cells (t8)–t4) [54]. The fertilization rate was also calculated as the number of fertilized eggs/number of injected eggs x100, considering an egg as fertilized when it ejects the second polar body and contains 2 pronuclei. In addition, we calculated the compaction rate (number of compacted embryos/number of fertilized eggs x 100) and the blastulation rate (number of blastocysts/number of fertilized eggs x 100) per cycle [40]. Blastocyst quality was evaluated according to the Gardner and Schoolcraft grading system using numerical grades for the degree of blastocyst expansion (1 = blastocoel less than half of the embryo 2 = blastocoel more than half of the embryo, 3 = blastocoel entirely filling the embryo, 4 = blastocoel larger than the embryo); trophectoderm (TE) (1 = many cells creating a cohesive layer, 2 = several cells forming a loose epithelium, 3 = very few large cells), and inner cell mass (ICM) quality (1 = many cells, tightly packed, 2 = several cells, loosely grouped, 3 = very few cells) [55].

### Statistical analysis

Results are expressed as mean ± standard deviation (SD) for normally distributed continuous variables, median ± interquartile range for non-normally distributed continuous variables, and as percentage where applicable. Statistical analysis was done using an R package (R Core team 2014). We used the module rbiostats that utilizes Student’s t-test and analysis of variance (ANOVA) with post-hoc tests (e.g., Tukey) in order to test for the statistical significance changes between two groups and among all of them, respectively [56, 57]. Since both tests require a normally distributed data, Shapiro-Wilk test was used to assess the normality of each group [58]. Moreover, Student’s t-test and the conventional ANOVA assume equal variances among control and independent variables. Hence, Bartlett’s equal of variance homogeneity test was performed [59]. When the data did not meet the normality and homogeneity requirements, a non-parametric Kruskal-Wallis test was applied for the analysis of statistical significance among groups. Spearman correlation analysis was performed between the 11 ordinary analysed variables (BMI, WC, % of sperm concentration, progressive and non-progressive motilities, typical morphology, % of DNA methylation and DNA hydroxymethylation and % of Ros^+^, AB^+^ and TB^+^ cells) because our data do not follow a Gaussian distribution. A p value <0.05 was considered statistically significant. Principal component analysis plots (PCA) were generated using the function Rcmdr in the R package.

## Results

### Characteristics of the cohort

In this study, male participants were divided into three groups according to their BMI and WC (Table 1): lean (18<BMI<25 kg/m^2^ and WC< 90 cm), overweight (25≤BMI<30 kg/m^2^ and 90 cm ≤WC<102 cm), and obese (BMI≥30 kg/m^2^ and WC ≥102 cm). There were no statistically significant differences between the groups for the analysed personal characteristics, lifestyle factors, and reproductive history (p>0.05) (Table 1).

**Table 1:** Characteristics of the study population according to the paternal BMI and WC.

Paternal characteristics		Lean (n = 40)	Overweight (n = 42)	Obese (n = 46)
Personal characteristics	Age (years)	35±8	38±7	39 ±8
	Sexual abstinence (days)	4±2	4±3	5±2
Anthropometric	Body Mass Index (BMI)(Kg/m^2^)	23.7±1.3	27.6±1.4***	34±5***^§^
measurements	Waist circumference (WC)(cm)	84.9±6	99.1±5.8***	113±12.6***^§^
Lifestyle (yes) (%)	Tobacco intake (%)(<21 cigarettes/day)	43.3	43.7	45.45
	Alcohol consumption (%)(<5 units/week)	10	9.37	8.82
Reproductive history	Varicocelectomy (yes), (%)	10.3	9.6	9
	Orchidopexy (yes) (%)	0	3.2	0
	Hydrocelectomy (yes), (%)	0	0	0
	Inguinal hernia surgery (yes), (%)	6.8	12.9	6,06
	Primary infertility (yes), (%)	72	77.4	78.78
	Secondary infertility (yes), (%)	27.58	22.58	21.21

Results are expressed as mean ± standard deviation (SD) for normally distributed continuous variables, median ± interquartile range for non-normally distributed continuous variables, and as percentage for discrete variables.

Stars denote groups that are statistically significantly different from the control group

* (p<0.05)

** (p<0.01)

*** (p<0.001).

^§^ Studied groups (Overweight and Obese) showing statistically significant difference between them (p<0.05).

### Impact of density gradient centrifugation and swim-up procedure on conventional sperm parameters according to paternal BMI and WC

In assisted reproductive technology, several sperm preparation techniques such as density gradient centrifugation and swim-up procedure could be used to separate motile sperm with good morphology from dead and abnormal forms of sperm, immature germ cells and non sperm cells. The advantages and disadvantages of each technique were evaluated in several reports without reaching a firm consensus [60] [49, 61]. Therefore, in the present report, both procedures were used to evaluate the effect of obesity on conventional parameters of motile spermatozoa-enriched population. Before processing, the raw semen of overweight and obese men presented a significantly lower sperm concentration (p<0.001 and p<0.01, respectively), lower percentage of spermatozoa with progressive motility (p<0.001 and p<0.001, respectively) and lower percentage of sperm with normal morphology (p<0.01 and p<0.001, respectively) when compared to the raw semen of lean men (Table 2).

**Table 2:** Comparison in conventional sperm parameters before and after semen processing in the different studied groups.

Sperm parameters		Concentration (x10^6^/ml)	Motility (%)					Dead cells (%)	Normal morphology (%)
			Progressive		Non-progressive		Non motile		
Raw semen	Lean	54(45)	35(7.45)	30(7.7)		35(16.25)		22.42(18)	10(0)
	Overweight	30(24.5)***	22.5(10)***	30(15)		48.85(20)***		27.05(19.62)	6(9.25)**
	Obese	37.5(36.25)**	22.5(5.87)***	25(10)		50(21.25)		27(21.25)	4(2)***
Density gradient technique	Lean	50 (45)	70(45)	15(20)		10(17.5)		10.5(14.75)	11(8.**)
	Overweight	20 (30) ***	71.8(47.5)	10(18.75)		16(28.75)		14.75(28.97)	3(7)**
	Obese	27.5 (38) **	64(35.75)	20(18.75)		12.5(21.37)		18(20.125)	4.5(6.25)***
Swim-up technique	Lean	30 (23.5)	90(18)	7.5(11.25)		5(10)		7.25(6.8)	10(7)
	Overweight	10 (17) **	80(50)	10(30)		0(10)		5.75(9.57)	7(11.5)
	Obese	12.5 (23.25) **	80(45.35)	13.75(47.5)		0(10)		4.5(7.79)	5(4)

Results are expressed as median (interquartile range) for non-normally distributed continuous variables. Superscripts

** (p<0.01) and

*** (p<0.001) stand for statistically significant differences between overweight/obese men and lean men in each group.

After processing, significantly lower sperm concentrations were obtained after density gradient and swim-up procedures in the overweight group (p<0.001) and obese group (p<0.01) compared to the lean group (Table 2). Curiously, the percentages of sperm with normal morphology were shown to be statistically different in the overweight (p<0.01) and obese (p<0.001) groups compared to the lean group only after density gradient centrifugation but not after swim-up procedure (Table 2). Finally, there were no statistically significant differences in sperm motility between the different groups after density gradient or after swim-up techniques (Table 2).

To sum up, there were no statistically significant differences in sperm motility and morphology between groups after swim-up procedure (Table 2).

### The drawbacks of obesity on the molecular parameters of motile spermatozoa

#### Alterations in sperm chromatin after density gradient and swim-up procedures in overweight and obese groups

**Fig 3:**
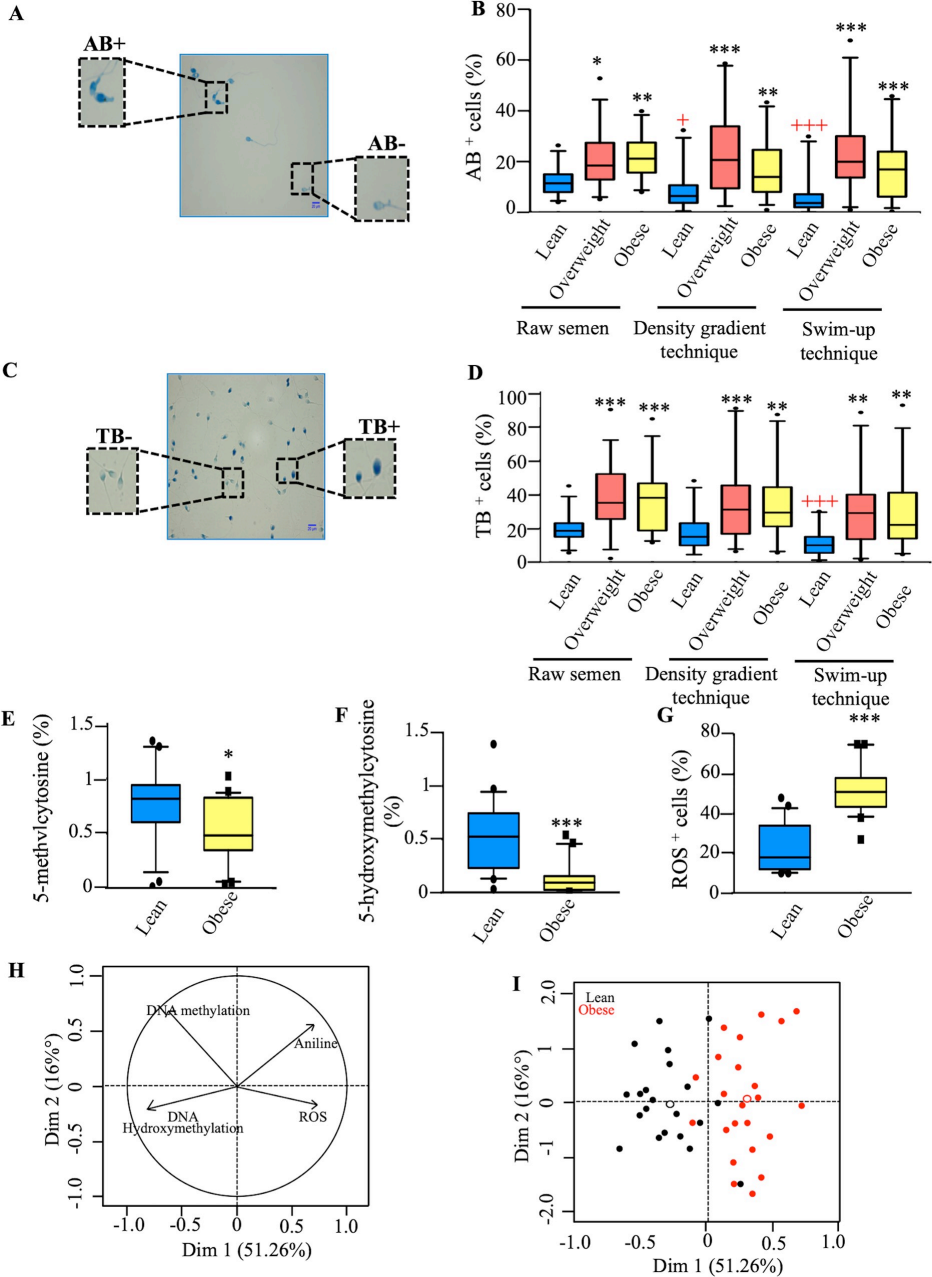
Alteration of sperm molecular parameters in obese men. (A, B) Histones retention as assessed by aniline blue staining (AB) in the sperm of lean, overweight, and obese men. (A) Light blue sperm heads show normal histones retention (AB^-^) and abnormal dark blue sperm heads show abnormal histones retention (AB^+^). Scale bar = 20 μm. Magnification x100. (B) Boxplots presenting the percentage of AB^+^ cells in raw semen, and in motile sperm-enriched fractions post- density gradient and swim-up techniques of the lean, overweight, and obese groups. (C, D) Chromatin integrity as assessed by the toluidine blue staining (TB) in the sperm of lean, overweight, and obese men. (C) Light blue sperm heads show normal chromatin integrity (TB^-^) and abnormal dark purple sperm heads show abnormal chromatin integrity (TB^+^). Scale bar = 20 μm. Magnification x100. (D) Boxplots presenting the percentage of TB^+^ cells in raw semen and in motile sperm-enriched fractions post-density gradient and swim-up techniques of the lean, overweight, and obese groups. Boxplots presenting the global sperm content of 5-methylcytosine (E) and 5-hydroxymethylcytosine (F) DNA methylation. (G) ROS level as assessed by nitroblue tetrazolium test in the motile sperm of obese and lean men. Data expressed as median (min-max). Stars indicated groups that are statistically significantly different to control, (*p<0.05, **p<0.01, ***p<0.001). + indicated motile-enriched fractions which are statistically significantly different compared to the raw semen (+p<0.05, +++p<0.001). (H, I) Principal component analysis (PCA) plots. PCA analysis of the obesity and control groups based on molecular parameter values (DNA methylation, 5-hydroxymethulcytosine, AB+ cells percent and ROS levels). Variable factor maps (H). Individual factor map (I). Red and black points indicate obese and lean men, respectively.

It is well described that paternal obesity can alter the sperm epigenetic program [17, 20, 23]. However, the impact of high paternal BMI on global epigenetic status of the motile sperm is not fully understood. To evaluate this effect, we first assessed sperm chromatin composition (Fig 3A and 3B) and sperm chromatin integrity (Fig 3C and 3D) using aniline blue (AB) and toluidine blue (TB) cytochemical stainings, respectively. These tests were performed on the raw semen and on the motile sperm-enriched fractions prepared either by density gradient or swim-up procedures.

On one hand, a statistically significant higher percentage of sperm with high histones retention (AB^+^ sperm cells) was observed in the raw semen of the overweight and obese groups as compared to the lean group (p<0.05 and p<0.01, respectively) (Fig 3B). A similar trend was observed using the motile sperm fractionated either through the post-density gradient or the post-swim-up procedure, with a significant higher percentage of AB^+^ cells in the overweight and obese groups compared to the lean group (Fig 3B). A significant decrease in the percentage of AB^+^ cells was detected after swim-up procedure in comparison to that of the raw semen, only in the lean group (p<0.001) (Fig 3B).

On the other hand, the percentage of TB^+^ sperm cells, which evidences sperm with altered chromatin integrity, was higher in the raw semen of the overweight and obese groups when compared to that of the lean group (p<0.001) (Fig 3D). Furthermore, the percentage of TB^+^ cells was significantly higher in the overweight and obese group, post-density gradient, compared to that of lean group (p<0.01 and p<0.01, respectively) (Fig 3D). Similar findings were detected after swim-up procedure, with a significant higher percentage of TB^+^ cells in the overweight and obese group compared to the lean group (p<0.01 and p<0.01, respectively) (Fig 3D). Only in the lean group, the swim-up procedure was able to decrease the level of TB^+^ cells compared to that of the raw semen (p<0.001) (Fig 3D).

Strikingly, while the percentages of AB^+^ sperm and TB^+^ sperm were correlated with the percentages of sperm with normal morphology in the raw semen (S1 Table), no correlation between these parameters was evidenced in the motile sperm-enriched fraction (R = 0.04; p>0.05 and R = -0.14; p>0.05, respectively) (S2 Table).

#### Alterations in the global DNA methylation and DNA hydroxymethylation status in the motile spermatozoa fraction of obese men

To further analyse the effect of obesity on molecular parameters of motile sperm, we assessed the global 5-methyl- and 5-hydroxymethyl-cytosine methylation status in motile sperm of obese and lean groups. These experiments were performed on the motile sperm derived from the swim-up procedure. Our results showed that the percentage of 5-methylcytosine was significantly lower in obese men compared to lean men (p<0.05) (Fig 3E). Of particular interest, the levels of 5-methylcytosine were negatively correlated with the percentage of the TB^+^ sperm cells (R = -0.34; p<0.05) (S1 Table). In addition, we observed a lower level of 5-hydroxymethylcytosine in obese men compared to lean men (p<0.001) (Fig 3F). As shown in S2 Table, 5-mC was negatively correlated with both WC (R = -0.39, p = 0.01) and BMI (R = -0.46, p = 0.01). Plus, 5-hmC was either negatively or positively correlated with BMI (R = 0.49, p = 0.001) and WC (R = -0.55, p = 0.001), respectively. Importantly, neither the levels of 5-mC nor the levels of 5-hmC were found correlated with the percentage of motile sperm normal morphology (S2 Table).

#### Higher percentage of ROS positive cells in the motile sperm of obese men compared to that of lean men

Knowing the strong correlation existing between obesity and systemic oxidative stress [14, 35, 62, 63], we aimed to compare the intracellular ROS levels in the motile sperm between lean and obese men. For this purpose, intracellular reactive oxygen species (ROS) levels were evaluated with the nitroblue tetrazolium test in the motile sperm derived from the swim-up procedure from obese and lean men. We observed an increased percentage of ROS^+^ cells in obese men’s spermatozoa compared to the lean men’s spermatozoa (p<0.001) (Fig 3G). Particularly, our results showed strong positive correlations between the percentage of ROS positive motile sperm and BMI values (R = 0.69, p<0.001), WC values (R = 0.67, p<0.001), the percentage of AB^+^ motile sperm (R = 0.3, p<0.01), and the percentage of TB^+^ motile sperm (R = 0.39, p<0.001) (S2 Table). Plus, negative correlations were detected between the percentage of ROS positive motile sperm and 5-mC levels (R = -0.36, p<0.05) and 5-hmC levels (R = -0.40, p<0.01) (S2 Table). In contrast, the percentage of motile spermatozoa with normal morphology was not correlated to the percentage of ROS positive cells (S2 Table). Altogether these results indicated that while there was no difference between groups in the conventional sperm parameters after swim-up, molecular alterations might still exist in the motile sperm with normal morphology

Based on these findings, we considered that molecular sperm parameters profiles would differ between obese men compared with lean men. Sperm molecular profiles comparison of these two groups was conducted and principal component analysis (PCA) was performed using 5-mC, 5-hmC and chromatin parameters (Fig 3H and 3I). Based on the sperm molecular profiles, good separation between the 2 groups can be observed in the PCA score plot (Fig 3I) with only few exceptions.

### Effect of paternal overweight and obesity on pre-implantation embryo morphokinetics

In the light of the above results showing that motile sperm are affected by obesity at the molecular level, and due to the role of sperm epigenome in early embryo development [64, 65], pre-implantation embryo morphokinetics as well as embryo quality were evaluated in the context of high paternal BMI and WC. These morphokinetic parameters of the developing embryos provide a strong indicator of the implantation and the ongoing pregnancy rates [66]. Embryos fertilized with sperm from either lean, overweight or obese men were monitored by time-lapse imaging. Maternal BMI, age, and number of MII oocytes/per cycle (p>0.05) were similar in the three groups as well as for the percentage of females with polycystic ovaries, endometriosis, and tubal/pelvic disease (p>0.05) (Table 3). The fertilization rate for ICSI was not significantly different between the lean, overweight and obese groups, being of 90.5, 74.28 and 84% (p>0.05), respectively.

**Table 3:** Characteristics of the female cohorts according to the paternal BMI and WC.

Maternal characteristics	Paternal characteristic		
	Lean (n = 10 couples)	Overweight (n = 10 couples)	Obese (n = 12 couples)
Body Mass Index (BMI) (kg/m^2^)	24.33±4.65	23.96±0.61	22.79±3.44
Age (years)	31±6.5	28.5±12	30±6.5
Number of MII oocytes/woman	6.5±9	6±3.75	6±4
Total number of MII oocytes	71	73	80
Fertilization rate (%)	90.5	74.28	84
Polycystic ovaries (%)	12.5	20	22
Endometriosis (%) (0 = No ; 1 = Yes)	0	11.1	0
Tubal/pelvic disease (%)(0 = No ; 1 = Yes)	0	20	0
Other (%)(0 = No ; 1 = Yes)	25	33.33	40
Previous attempts	1.5 ± 1.69	2.3 ± 2.11	2.36 ± 2.54

Results are expressed as median ± interquartile range for non-normally distributed continuous variables and as percentage for discrete variables. There is no statistically significant difference between groups.

**Fig 4:**
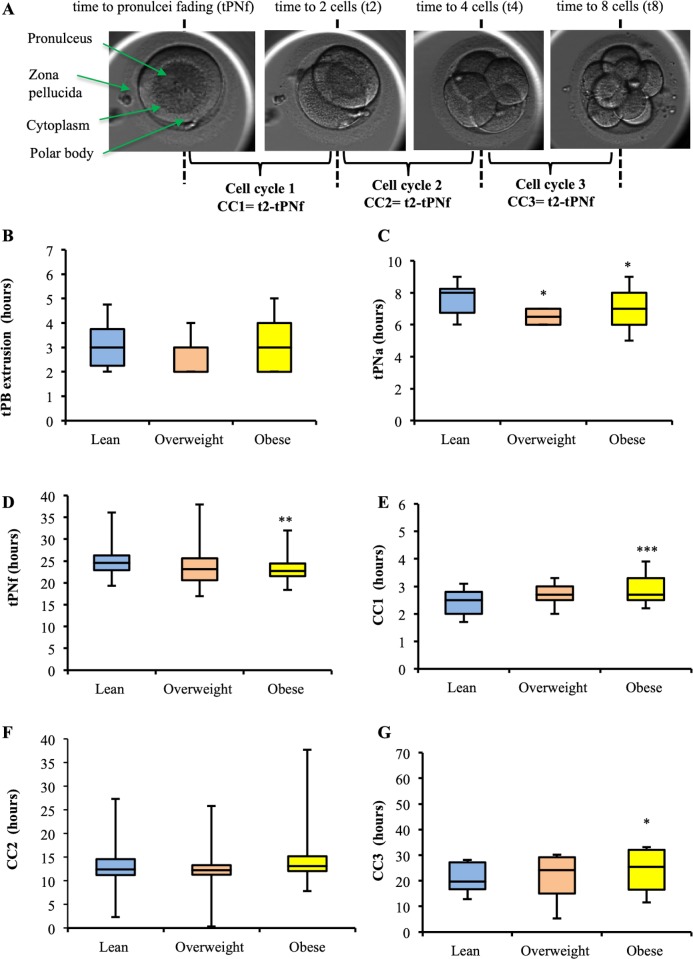
Effect of obesity on the morphokinetic parameters of pre-implantation embryo development. (A) Pictures of pre-implantation embryo development. Boxplots presenting the results expressed as median ± interquartile range for (B) the time of the second polar body extrusion (tPB extrusion), (C) the time when the first pronuclei is condensed and visible (pronuclei appearance, tPNa) (D), the time when both pronuclei disappear (tPNf) (E), the length of the first embryonic cell cycle (CC1 = time to 2 cells (t2)-(tPNf), (F) the length of the second embryonic cell cycle (CC2 = time to 4 cells (t4)-(t2)), and (G) the length of the third embryonic cell cycle (CC3 = time to 8 cells (t8)-(t4)) in the embryos derived from normal (blue boxplots), overweight (pink boxplots), and obese (yellow boxplots) fathers. Stars indicated groups that are statistically significantly different from the lean group (*p<0.05, **p<0.01, ***p<0.001).

**Fig 5:**
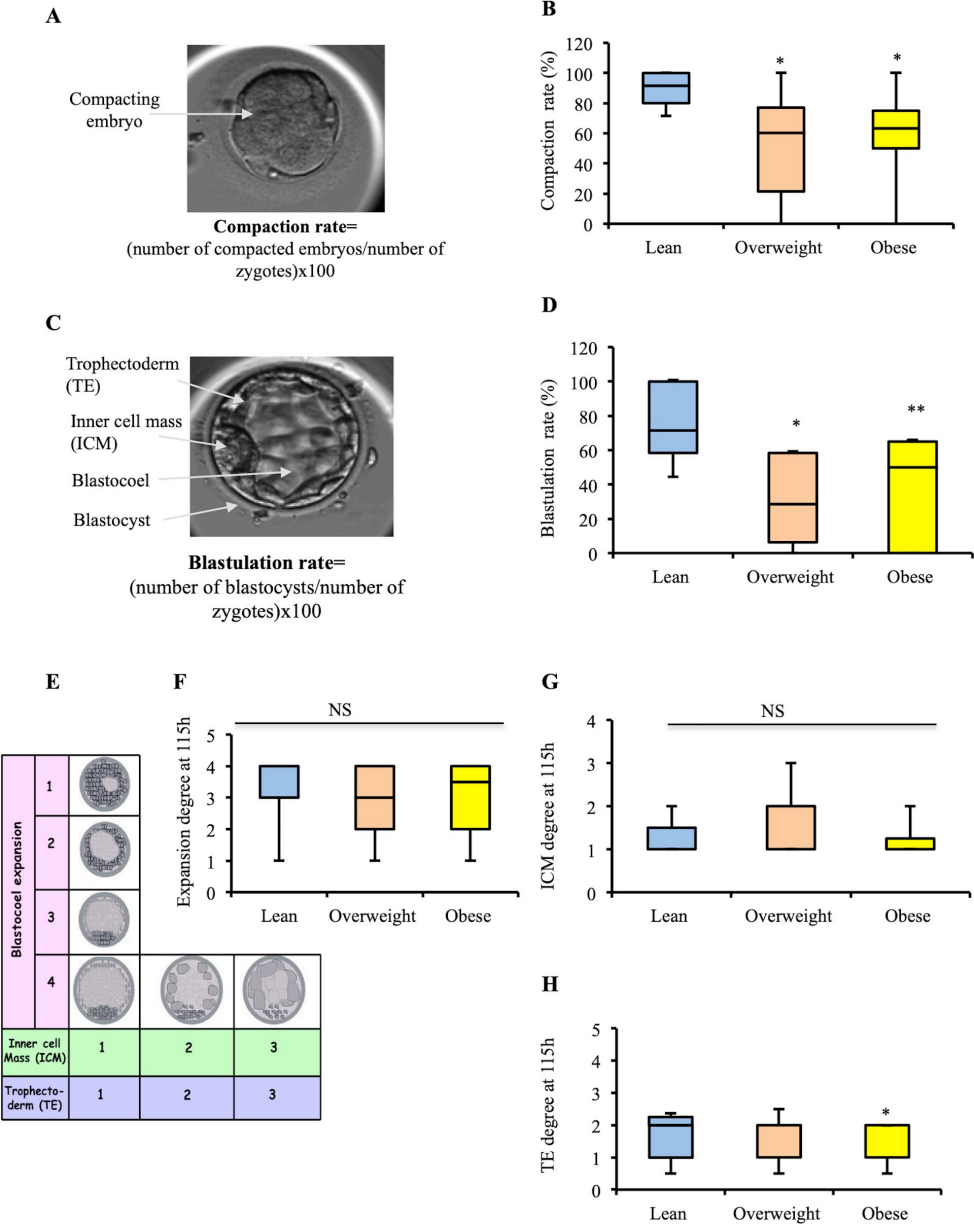
Effect of paternal obesity on the embryo development on day 4 and 5 of culture. (A) Picture of a compacting embryo. (B) Boxplots showing the embryo compaction rate in the different groups. (C) Picture of a blastocyst. (D) Boxplots showing the blastulation rate in the three different groups. (E) Blastocyst grading system used (expansion degree from 1 to 4, inner cell mass (ICM) quality from 1 to 3, and trophectoderm (TE) quality from 1 to 3). (F) Boxplots showing the degree of blastocoel expansion at 115h in the different groups. (G) Boxplots showing the ICM quality at 115 h in the three different groups. (H) Boxplots showing the TE quality at 115h in the different groups. Data are expressed as median ± interquartile range. Stars indicated groups that are statistically significantly different from the lean group (*p<0.05, **p<0.01, ***p<0.001).

As shown in Fig 4, several morphokinetic parameters were statistically significantly affected by paternal overweight and obesity. At first, although the time of second polar body extrusion was not affected by paternal obesity (Fig 4B), the times of pronuclei appearance (tPNa) (Fig 4C) and pronuclei fading (tPNf) (Fig 4D) were shorter in the obese group (p<0.05 and p<0.01, respectively) as compared to the lean group. Furthermore, the duration of the first embryonic cell cycle (CC1) was longer in the obese group than that of the CC1 in the lean group (p<0.0001) (Fig 4E). However, there was no statistically significant difference in duration of the second embryonic cell cycle (CC2) between the three groups (Fig 4F). In contrast, the duration of the third embryonic cell cycle (CC3) was significantly delayed in the obese group compared to the lean one (p<0.05) (Fig 4G). In addition to these early embryonic alterations, the compaction and blastulation rates were statistically significantly affected by high paternal BMI and WC (Fig 5A–5D). Particularly, the compaction rate was significantly lower in the overweight and obese groups compared to the lean group (p<0.05 and p<0.05, respectively). Regarding the blastulation rate, it was significantly lower in overweight and obese groups compared to the lean group (p<0.05 and p<0.01, respectively).

To establish whether paternal obesity may affect the blastocyst quality, the grades of blastocysts at day 5 (blastocyst expansion, trophectoderm and inner cell mass morphologies) were scored as previously described (Balaban et al. 2011) (Fig 5E). There were no statistically significant differences in the blastocyst expansion degree (Fig 5F), and in the inner cell mass (ICM) morphology across the groups (Fig 5G). In contrast, a significant higher score for the trophectoderm (TE) quality was detected in the obese group compared to lean group (p<0.05) (Fig 5H). According to the scoring system used in the study, a high score for TE (>1) refers to a lower embryo quality.

## Discussion

Our work showed that paternal obesity (as measured by BMI and WC) is associated with alterations in the molecular composition of the motile sperm-enriched fraction as well as in pre-implantation embryo morphokinetics.

We selected 88 overweight/obese and 40 lean men to evaluate the impact of obesity on conventional semen parameters, on various characteristics of motile sperm prepared by two different sperm preparation techniques, and on embryo morphokinetics. Regarding the raw semen, our results clearly support the observation of others that conventional semen parameters are affected by high BMI and could subsequently decrease the male fertility potential (review in [11, 14, 67]).

During infertility treatment with ICSI, a morphologically normal motile spermatozoon is selected for microinjection into a mature oocyte, when possible. However, sperm motility and morphology do not always reflect the molecular composition of the spermatozoa [31]. Therefore, we aimed to assess the impact of paternal obesity on the molecular composition of motile spermatozoa that could be used during ICSI. In order to avoid any potential technical bias, each semen sample was divided into aliquots and each aliquot was either fractionated with density gradient centrifugation or swim-up technique. These two sperm preparation methods are widely used in ART and each method has its own advantages and disadvantages [49, 68, 69].

Our analyses revealed no statistically significant differences in sperm motility between lean, overweight and obese groups, after density gradient centrifugation and swim-up procedure. Moreover, there were no statistically significant differences in the percentages of spermatozoa with normal morphology between the three analysed groups, after swim-up procedure. By contrast, after density gradient and swim-up techniques, we detected a statistically significant higher percentage of motile sperm with altered chromatin in obese and overweight men compared to lean men, confirming their susceptibility to biological insults, such as obesity [20, 23]. Of particular interest, the percentage of spermatozoa bearing chromatin lesions was not significantly correlated to the percentage of sperm with normal morphology, supporting the idea that sperm motility and morphology per se are not strong predictors of chromatin quality in obese men [31].

In parallel, we evaluated the global 5-mC and 5-hmC contents, in the motile sperm-enriched fraction post swim-up technique. 5-mC and 5-hmC are epigenetic marks globally associated either with gene repression or active gene transcription, respectively [70, 71]. The levels of these two epigenetic marks were significantly lower in sperm of obese participants than in that of lean men. We also found a negative correlation between the sperm 5-mC DNA methylation and the altered chromatin integrity and between 5-hmC levels and sperm histone retention. Moreover, 5-mC levels were positively correlated to the 5-hmC levels in the motile spermatozoa. Importantly, percentages of motile sperm with normal morphology were not correlated to the 5-mC and 5-hmC levels. It is apparent, and not surprising in the light of previous studies, that the 5-mC levels were altered in sperm of obese participants [20, 23]. Indeed, associations between 5-mC alteration and fertility have been suggested in many occasions. Thus, several studies have manifested that global sperm DNA methylation was lower in participants with high seminal reactive oxygen species (ROS) and with spermatozoa that have high DNA fragmentation index, abnormal chromatin condensation, and those with reduced motility [72, 73]. Our results showed higher levels of ROS in the sperm of obese men compared to that of lean men. This could be one of the reasons behind the global DNA hypomethylation found in this studied group compared to the control group[20, 23, 74]. In parallel, Oakes et al. have demonstrated that male mice treated with DNA methylation inhibitor drug, suffered from reduced sperm motility and the inability of the demethylated paternal genome to support embryo development [75].

Moreover, here we provided the first report, to our knowledge, showing the significant low level of 5-hmC in sperm of obese participants compared to lean participants. One possible explanation of this finding could be the low level of 5-mC available for conversion to 5-hmC in the spermatozoa of obese men compared to the lean men. In addition, our results are in line with several studies showing that the environmental exposure may affect the DNA hydroxymethylation profile in the somatic cells. For instance, it was demonstrated that the exposure to oxidative stress leads to a decrease in the global 5-hydroxymethylcytosine patterns *in vivo* and *in vitro* [76–78]. In this context, we found a significant negative correlation between ROS positive sperm and the level of global DNA hydroxymethylcytosine. In light of these data, it will be important to study the molecular mechanisms by which obesity modifies the sperm global DNA hydroxymethylation pattern, identify the directly modified loci through genome-wide epigenetic analysis, and finally examine the potential effect of these alterations on embryogenesis.

Therefore, we may conclude that obesity in fathers exerts a negative influence on the molecular components of the motile spermatozoa. It is known that sperm chromatin condensation is important to protect sperm DNA during its transit in the female reproductive tract and also to control epigenetic reprogramming during the pre-implantation period [74, 79]. Chromatin condensation and DNA integrity are often correlated with poor fertility outcomes which are generally characterized by low fertilization rates, poor embryo quality, repeated failures of assisted reproductive technology attempts, and miscarriages [72, 73, 75, 80]. However, we did not evidence a significant difference between the fertilization rates of the 3 groups that may be associated with the characteristics of our cohort (infertile patients and small sample). Despite this fact and thanks to assessment of the developmental capacity of cultured embryos derived from obese fathers in a time-lapse incubator, we revealed a negative impact of a high BMI/high WC on early embryo development. Indeed, we observed an earlier PN appearance and earlier PN fading in the obese group compared to the lean group. Early PN fading was shown to be associated with a decrease in live birth rate [81]. We also found that paternal overweight and obesity were significantly associated with prolonged durations of the first and third embryonic cell cycles and with decreased rates of compaction and blastulation. Considering that delayed cleavage intervals have been associated with a lower blastocyst expansion rate and a lower implantation rate [66, 82], it may, therefore, be assumed that a high BMI/high WC have unfavorable effects on developmental ability of human embryos.

Altogether, our data provide additional evidence that paternal obesity may contribute to the decline of male fertility observed over the world. The molecular mechanisms involved in this process are far from being understood at present. It is known that after fertilization, the sperm genome undergoes highly-hierarchial epigenetic modifications involving removal of sperm nuclear envelope, decondensation of the chromatin through the reduction of the disulfide bonds between protamines, replacement of paternal protamines by maternal histones, and reprogramming DNA methylation [83]. Thus, we can hypothesize that any slight alteration in one of the sperm profiles will have detrimental consequences on embryo development and subsequently on adult health [84].

## Conclusions

Our data indicate that male obesity is associated with various epigenetic modifications in the motile sperm and with distinct morphokinetic changes in the preimplantation embryos. Further studies are needed to validate on one hand whether a high BMI-induced sperm epigenetic signature modifications are associated with abnormal embryonic development, and on another hand whether embryo morphokinetics alterations cause long-term health hazards on neonates. Considering the interplay between obesity, molecular sperm quality and embryonic development, our data suggest that the time-lapse imaging technology might be considered in a future as an important tool enabling a better selection embryo in the context of pathologies known to alter the sperm quality.

## Supporting information

S1 Table Correlations between BMI, WC, conventional and molecular sperm parameters in raw semen statistically significant correlation (*p<0.05, **p<0.01, ***p<0.001). (DOCX)

S2 Table Correlations between BMI, WC, conventional and molecular sperm parameters in the motile sperm–enriched fraction. (DOCX)

## Abbreviations

ART: assisted reproductive technology
BMI: body mass index
5-hmC: DNA hydroxymethylation
5-mC: DNA methylation
CC1: first cell cycle
CC2: second cell cycle
CC3: third cell cycle
hCG: human chorionic gonadotrophin
ICM: inner cell mass
ncRNAs: noncoding RNAs
NBT: nitroblue tetrazolium test
ROS: reactive oxygen species
tPNa: timing of pronuclei appearance
tPNf: timing of pronuclei fading
TB: toluidine blue
TE: trophectoderm
T2DM: type 2 diabetes mellitus
WC: waist circumference
