# Supplementary material for: Adverse effects of paternal obesity on the motile spermatozoa quality
Source: PLoS One. 2019 Feb 11;14(2):e0211837. doi: 10.1371/journal.pone.0211837 (PMC6370200; doi:10.1371/journal.pone.0211837)
Supplement: S1 Table — (DOCX) [file pone.0211837.s001.docx]

**S1 Table**: Correlations between BMI, WC, conventional and molecular sperm parameters in raw semen

|  | 1 | 2 | | | 3 | 4 | | 5 | | 6 | | 7 | | |
| --- | --- | --- | --- | --- | --- | --- | --- | --- | --- | --- | --- | --- | --- | --- |
| 1. BMI |  | |  |  | | |  | |  | |  | |  |  |
| 2. WC | **0.89***** | |  |  | | |  | |  | |  | |  |  |
| 3. Concentration | -0.19 | | -0.21 |  | | |  | |  | |  | |  |  |
| 4. Progressive motility | **-0.34*** | | **-0.36*** | **0.56***** | | |  | |  | |  | |  |  |
| 5. Non-progressive | **-0.29*** | | **0.29*** | **0.31**** | | | **0.51***** | |  | |  | |  |  |
| 6. Typical morphology | **-0.38***** | | **-0.43***** | **0.31**** | | | **0.42***** | | 0.22 | |  | |  |  |
| 7. Aniline (AB^+^) | **0.39***** | | **0.39***** | **-0.35**** | | | **-0.38**** | | **-0.32**** | | **-0.41***** | |  |  |
| 8. Toluidine (TB^+^) | **0.56**** | | **0.58***** | **-0.36**** | | | **-0.38**** | | **-0.32**** | | **-0.37**** | | **0.44***** |  |

Stars indicated statistically significant correlation (*p<0.05, **p<0.01, ***p<0.001).
