# Supplementary material for: Adverse effects of paternal obesity on the motile spermatozoa quality
Source: PLoS One. 2019 Feb 11;14(2):e0211837. doi: 10.1371/journal.pone.0211837 (PMC6370200; doi:10.1371/journal.pone.0211837)
Supplement: S2 Table — (DOCX) [file pone.0211837.s002.docx]

**S2 Table**: Correlations between BMI, WC, conventional and molecular sperm parameters in the motile sperm–enriched fraction

|  | 1 | 2 | 3 | 4 | 5 | 6 | | 7 | | 8 | 9 | | 10 | |
| --- | --- | --- | --- | --- | --- | --- | --- | --- | --- | --- | --- | --- | --- | --- |
| 1. BMI |  |  |  |  |  |  | |  | |  |  | |  | |
| 2. WC | **0.89***** |  |  |  |  |  | |  | |  |  | |  | |
| 3. Concentration | -0.14 | -0.1 |  |  |  |  | |  | |  |  | |  | |
| 4. Progressive motility | **-0.26*** | -0.21 | **0.27*** |  |  |  | |  | |  |  | |  | |
| 5. Non-progressive | **0.29*** | **0.26*** | **-0.29*** | **-0.88***** |  |  |  | |  | | |  | |  |
| 6. Typical morphology | 0.04 | 0.07 | **0.24*** | **0.27*** | **-0.31***** |  |  | |  | | |  | |  |
| 7. 5-mC | **-0.46**** | **-0.39**** | 0.29 | 0.23 | **-0.18*** | -0.062 |  | |  | | |  | |  |
| 8. 5-hmC | **-0.49***** | **-0.55***** | 0.22 | 0.11 | -0.12 | 0.182 | **0.35*** | |  | | |  | |  |
| 9. Ros+ | **0.69***** | **0.67***** | -0.27 | **-0.29*** | **0.27*** | -0.11 | **-0.36*** | | **-0.40**** | | |  | |  |
| 10. Aniline (AB^+^) | 0.19 | 0.23 | **-0.31*** | -0.14 | 0.1 | 0.04 | -0.19 | | **-0.49***** | | | **0.3**** | |  |
| 11. Toluidine (TB^+^) | **0.33**** | **0.33**** | **-0.36*** | **0.43***** | **0.5***** | -0.14 | **-0.34*** | | **-0 .3*** | | | **0.39***** | | 0.19 |

Stars indicated statistically significant correlation (*p<0.05, **p<0.01, ***p<0.001).
